# Post-analytical laboratory work: national recommendations from the Working Group for Post-analytics on behalf of the Croatian Society of Medical Biochemistry and Laboratory Medicine

**DOI:** 10.11613/BM.2019.020502

**Published:** 2019-06-15

**Authors:** Jasna Lenicek Krleza, Lorena Honovic, Jelena Vlasic Tanaskovic, Sonja Podolar, Vladimira Rimac, Anja Jokic

**Affiliations:** 1 Working Group for Post-analytics, Croatian Society of Medical Biochemistry and Laboratory Medicine, Zagreb, Croatia; 2 Department of Laboratory Diagnostics, Children’s Hospital Zagreb, Zagreb, Croatia; 3 Department of Laboratory Diagnostics, General Hospital Pula, Pula, Croatia; 4 Medical Biochemistry Laboratory, General Hospital "Dr. Tomislav Bardek", Koprivnica, Croatia; 5 Department of Transfusion Medicine and Transplantation Biology, University Hospital Centre Zagreb, Zagreb, Croatia; 6 Department of Medical Biochemistry, Haematology and Coagulation, University Hospital for Infectious Diseases “Dr. Fran Mihaljević”, Zagreb, Croatia

**Keywords:** recommendations, post-analytical phase, clinical laboratory, harmonization, test report

## Abstract

The post-analytical phase is the final phase of the total testing process and involves evaluation of laboratory test results; release of test results in a timely manner to appropriate individuals, particularly critical results; and modification, annotation or revocation of results as necessary to support clinical decision-making. Here we present a series of recommendations for post-analytical best practices, tailored to medical biochemistry laboratories in Croatia, which are intended to ensure alignment with national and international norms and guidelines. Implementation of the national recommendations is illustrated through several examples.

## Introduction

The post-analytical phase is the final phase of laboratory work in which laboratory results are evaluated until they are released. The frequency of laboratory errors during the post-analytical phase is lower than the frequency of errors during the pre-analytical phase, yet the post-analytical phase accounts for nearly one quarter of the entire laboratory process (*1*-*6*). All laboratory personnel may be involved in the post-analytical phase in accordance with their competencies (*7*). The post-analytical phase can be further divided into a phase inside the laboratory and a phase outside the laboratory (post-post-analytical phase). The post-post-analytical phase is not covered in the present recommendations and refers to procedures in which a physician makes medical decisions based on laboratory test reports in order to provide timely and effective patient care (*8*).

## Recommendations

The present recommendations for the post-analytical phase were developed by the Working Group for Post-analytics of the Committee for Scientific Professional Development of the Croatian Society of Medical Biochemistry and Laboratory Medicine (CSMBLM). The recommendations are intended for laboratory experts who are responsible for timely and accurate release of laboratory test results. Such experts are mandated by regulations concerning the medical biochemistry profession to hold master’s degrees or specialisations in medical biochemistry and laboratory medicine, they must have passed the relevant board certification exam, and they must possess a valid license to practise from the Croatian Chamber of Medical Biochemists (CCMB). These experts are authorised to evaluate and release laboratory test results (*9*, *10*). These recommendations are based on CCMB regulations and recommendations, the International Organization for Standardization (ISO) 15189:2012 (Medical laboratories - Requirements for quality and competence), other national recommendations of the CSMBLM, laws and policies of the Republic of Croatia and recent literature (*7*). In addition, the recommendations are aligned with specific requirements of the medical biochemistry profession at the national level in the Republic of Croatia.

The aim of these recommendations is to encourage the implementation of certain procedures to simplify and harmonise the post-analytical phase of laboratory work. The most significant procedures are explained in the text together with relevant recommendations. Examples of how to implement the recommendations for certain situations are provided in the Appendices.

**Figure 1:**
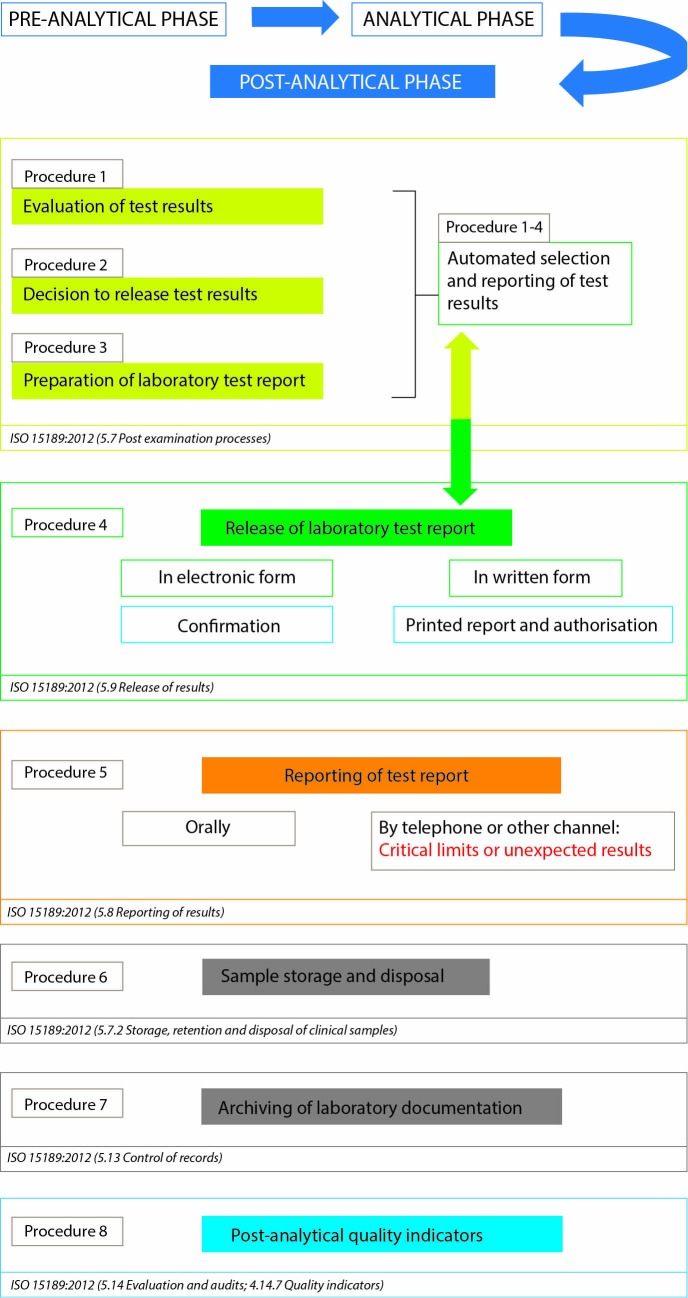
Procedures in the post-analytical phase of clinical laboratory work

The procedures of the post-analytical phase include (Figure 1):

1. Evaluation of test results
2. Decision to release test results
3. Preparation of the laboratory test report
4. Release of the laboratory test report
5. Reporting of test results
6. Sample storage and disposal
7. Archiving of laboratory documentation
8. Post-analytical quality indicators.

All procedures for the post-analytical phase are an integral part of ISO 15189:2012, thereby allowing rigorous quality control of post-analytical laboratory work (*7*).

For procedures not clearly defined by CCMB, our recommendations have been formulated based on the literature specified. In cases where the literature does not provide a clear viewpoint, the recommended procedures have been defined based on the consensus opinion of the Working Group.

Numerous national and international experts have reviewed this document, which was revised according to their valuable suggestions. Furthermore, the final version of the recommendations includes comments and suggestions from individual laboratory medicine specialists involved in public discussion as well as suggestions and approval from CCMB experts.

## PROCEDURE 1: Evaluation of test results

**Recommendation 1**

**All test results before release must be evaluated through two mutually independent activities: a) review of test results, which includes comparison of the results with reference intervals and/or critical results, patient diagnosis and previous test results (if available); and b) confirmation of test results.**

This is the first step in the post-analytical phase of laboratory work. All test results that are not confirmed and released immediately upon analysis as part of the automated selection and reporting of test results must be evaluated through two mutually independent activities: review and confirmation of test results.

The review of test results begins by comparing the results with reference intervals and/or critical results, diagnoses and previous test results, if available. After this comparison, the results are confirmed as acceptable, or additional procedures are recommended, such as repeating the test with remark results from device, diluting the sample if the results fall outside the measuring range, or confirming unexpected results using the same or a new sample. If additional procedures give unacceptable results, the laboratory test report is released (Procedure 2) without the unacceptable (controversial) result, together with an explanation in the “Comments” area about why the test results are invalid and what further procedures are recommended (Procedure 3).

The review of results in the post-analytical phase may reveal mistakes or new problems in both the pre-analytical and analytical phases (such as sample misidentification, which is part of the pre-analytical phase but is very often recognised post-analytically). The transition between the analytical and post-analytical phases of laboratory work depends largely on the particularities of the laboratory (size, personnel, instrumentation/middleware, and capabilities of the laboratory information system (LIS)). Thus, every additional effort should be made to assure quality of results.

## 1.1. Comparison with reference intervals

**Recommendation 2**

**Reference intervals or relevant limits for clinical decision-making according to age and gender have to be present next to each test result and are mandatory for the laboratory test report.**

**In the absence of a reference interval or when a reference interval stated is not specified in a national harmonisation document, this must be indicated in the “Comments” area as explained in ****Appendix 1****.**

Reliable reference intervals are an integral part of clinical interpretation of laboratory analyses. Each laboratory must define biological reference intervals or relevant limits for clinical decision-making cut-off values whenever possible. Document C28-A3 of the Clinical and Laboratory Standards Institute (CLSI) recommends the use of acceptable reference intervals regardless of their origin, which may come from the test reagent manufacturers, multi-centre studies, recommendations of regulatory bodies, medical literature and prevailing practice (*11*-*13*). Laboratory test results cannot be released without reference intervals (*14*). The absence of a reference interval or the use of a reference interval not recommended by a national harmonisation document (*e.g.* from a different source in the literature) must be indicated in the “Comments” area as explained in Appendix 1.

The reference interval of the population does not necessarily represent the reference intervals of individuals within the population. The index of individuality can estimate the usefulness of the reference interval as described in the next chapter (1.2.1. Reference change value).

Traceable, multi-centre reference intervals can be established after standardisation of the recommended analytical methods. Such reference intervals can be applied in all laboratories using methods traceable to reference measurement systems implemented under standardised pre-analytical conditions and applied to a population with socio-demographic and ethnic characteristics similar to those of the proband (*13*).

The most commonly used and recommended type of reference interval is defined as the central 95% interval bounded by the 2.5th and 97.5th percentiles for a reference population. These percentiles denote boundary values, and reference intervals include values between lower and upper limit values, including the limit values themselves. This means that 2.5% of individuals with the lowest values and 2.5% of individuals with the highest values are excluded from the reference distribution (*15*).

The need to apply laboratory test results rationally for all age groups and the need to harmonise results obtained using different analytical methods are driving national efforts to harmonise laboratory test results. In Croatia, the current CCMB document “Harmonisation of Laboratory Results in the Field of General, Specialist and Highly Differentiated Medical Biochemistry” describes the reporting of laboratory test results. Since 1 January 2005, all medical biochemistry laboratories (MBLs) in Croatia are required to use the recommended analytical methods from this CCMB document as a prerequisite for using the recommended reference intervals (*16*). For tests not covered by this document, MBLs usually apply reference intervals that are specified by test reagent manufacturers and verified on the population served by the laboratory (*17*).

In addition to comparing the reference interval, the CCMB document compares certain results to recommended standards (*e.g.* lipids), therapeutic intervals (*e.g.* drug monitoring during therapy), toxic concentrations (*e.g.* organic solvents, drugs of abuse), and limit values (“cut off” values, *e.g.* pleural effusion, addiction).

If reference intervals have not been determined for specific age groups, such intervals should not be interpolated or extrapolated from data from other age groups in the absence of studies based on the relevant analytical method. Given the challenge of properly determining reference intervals in paediatric populations, multi-centre data on reference intervals are typically taken from the literature. To be valid, the intervals for paediatric populations should be determined using an analytical method recommended by the CCMB and, if possible, verified on the target population (*18*, *19*). Several initiatives are developing databases of reference intervals for paediatric populations, including the Canadian Laboratory Initiative on Pediatric Reference Intervals (CALIPER), Nordic Reference Intervals Project (NORIP), German Health Interview and Examination Survey for Children and Adolescents (KiGGS), Children’s Health Improvement through Laboratory Diagnostics (CHILDx), and Harmonizing Age Pathology Parameters in Kids (HAPPI Kids) (*19*-*24*).

Instead of reference intervals, clinical decision limits can be used, particularly for tests that play a central role in decision-making involving a specific disease or condition for which cut off/decision limits have been established. In routine laboratory work, reference intervals are preferred over clinical decision limits because some tests have different cut-offs for different clinical conditions, such that unique clinical decision limits are in applicable. Furthermore, clinical decision limits may be used only if national or international guidelines have been established and implemented by physicians using the laboratory. The “Comments” area on the laboratory test report should clearly indicate that a clinical decision limit has been used for a given test (*12*).

### 1.2. Comparison with previous results

**Recommendation 3**

It is recommended that the review of test results include the testing of the difference between two consecutive results (delta check) whenever a predetermined result exists, because any difference between successive results that exceeds the defined limits may indicate (a) a significant change in the patient’s clinical condition, or (b) a problem with the sample.

#### 1.2.1. Reference change value

The reference interval is the primary data source used in the interpretation of laboratory results. However, when the index of individuality of an analyte is smaller than 1.4, especially less than 0.6, a reference interval is less useful because even though a given result is located within the reference range for an individual, even a small change in the result can indicate a clinically significant change (*3*). In these cases, it is useful to assess the significant change in serial results from one individual using the reference change value (RCV). Reference change value is calculated from the formula:

RCV (%) = 2^1/2^ x *Z* x (CV_A_^2^ + CV_I_^2^)^1/2^,

where Z is the number of standard deviations appropriate to the probability [Z = 1.96 for a 95% confidence interval (P < 0.05) and 2.58 for a 99% confidence interval (P < 0.01)], CV_A_ is the analytical imprecision that each laboratory calculates from its own internal quality control data, and CV_I_ is within-subject biological variation (*25*, *26*). Reference change value is most commonly used as a delta check value, especially in automated algorithms for selection and reporting of laboratory test results (*27*).

#### 1.2.2. Testing the difference between two consecutive results (delta check)

**Recommendation 4**

A delta check should be used as an integral part of the LIS with automatic alerts, indicating when the result exceeds the pre-defined limits of the delta check, as described in Appendix 2. The difference from the previous result is calculated as a delta percent change and compared with RCV limits.

A delta check assesses the difference between two consecutive test results for a certain analyte in the same patient with clearly defined criteria. Any difference between successive results that exceeds the defined limits may indicate (a) a significant change in the patient’s clinical condition, or (b) a problem with the sample. Problems with the sample may reflect errors in laboratory procedures that were not identified in earlier quality control procedures, such as sample mismatch or miss-identification, sample contamination with intravenous (IV) fluid, improper sample acquisition or handling (insufficient sample volume, clotted sample), or poor sample quality (haemolysis, lipemia, icterus) (*28*). It is also known that some analytes are generally more useful than others when performing delta checks. An example of such an analyte is alkaline phosphatase or mean corpuscular volume (MCV). These analytes have little day-to-day variation, low RCV and low index of individuality. The index of individuality corresponds to the ratio of CVi and inter-subject biological variation CVg. If an analyte’s index of individuality is < 0.6, then any shift in values indicates a change in a patient’s clinical status, even if the results lie within the reference interval (*28*).

Table 1 illustrates methods for calculating the difference between consecutive measurements (*25*, *29*, *30*). Laboratories should define their own limits beyond which delta check values are considered significant; these limits should be defined according to the patient population, type of laboratory test and existing clinical recommendations. The acceptability and applicability of the limits defined for delta check should be verified periodically (*31*-*35*). Delta check values can be expressed as percentages and compared to the RCV. It is also possible to modify the delta check formula to take into account how much time elapsed between the consecutive test results (*29*-*32*). The recommended interval between measurements is 2-5 days, though laboratories should define an acceptable interval based on their own population. Patients visit primary health care (PHC) facilities much less frequently than hospital laboratories, yet patients in PHC laboratories often have more stable clinical status. Appendix 2 shows examples of performing the delta check.

**Table 1:** Methods of performing the delta check between consecutive measurements

**Method**	**Equation**
Delta difference	Current value – previous value
Delta percent change	[(Current value - previous value) / previous value] x100
Rate difference	Delta difference / delta time
Rate percent change	Delta percent change / delta time

Considering that the delta check is a post-analytical method that assures that pre-analytical error does not lead to false laboratory results, laboratories should define methods to perform the delta check as well as actions to be taken when the delta check exceeds the laboratory-specified limits. These actions should exclude all possible sources of poor quality of results. Recommended actions are presented in Recommendation 5 (*36*).

**Recommendation 5**

Recommended actions when the delta check exceeds the laboratory-specified limits include:

a) reviewing clinical data (clinical diagnosis, therapeutic interventions, contacting a physician);

b) retesting the current and previous sample (if available), including primary tubes and aliquots;

c) checking for the presence of haemolysis, lipemia, icterus, clot or error in tube labelling of the previous and current sample, including primary tubes and aliquots; and

d) if all previous actions taken to find a source of the observed difference in results do not provide a valid explanation for such a difference, the analytical system must be re-checked for proper functioning.

In a LIS, automatic alerts can be set up to indicate when the result of a delta check exceeds pre-defined limits and how many days can elapse between consecutive measurements before a delta check is no longer valid. In this way, the LIS can support the delta check as part of the automated algorithm for selection and reporting of test results. Even in the absence of such automatic alerts, the LIS facilitates manual, subjective comparison of new results with previous ones for the same analyte. Such a comparison should consider how much time elapsed between the two measurements being compared, the patient’s clinical diagnosis and therapy history, pre-analytical variables and analyte variability. If a difference between two measurements is suspected to be due to patient or sample miss-identification, evaluation of results and any further actions should take into account all the patient’s existing samples.

### 1.3. Additional procedures

Additional procedures may be required to analyse samples whose results do not satisfy pre-defined laboratory criteria according to good laboratory practice. Additional procedures in the analytical phase may be needed to obtain reliable results. These procedures are usually triggered by defined limits implemented in middleware or the LIS. In other words, the post-analytical phase allows us to monitor performance of the analytical phase. The most common additional procedures are described below.

#### 1.3.1. Sample dilution

**Recommendation 6**

When results exceed the upper limit of the analytical measurement range, automatic dilution by the analyser should be used if possible. Manual dilution must follow the instructions of the reagent manufacturer. The laboratory must define the reportable analytical range for each test, as well as maximum allowable dilution.

The laboratory must define and examine the dilution protocol according to the measurement procedure. It must define the range of reporting results, including the analytical measurement range and maximum dilution that can be used for each test, as well as the method for which dilution is applicable.

Analysers are programmed with an analytical measurement range (AMR) to ensure that their results are valid, and if a result exceeds the upper limit of the AMR, the sample may need to be diluted to bring its results within the AMR. Many analysers feature automatic sample dilution (auto dilution) for this purpose; if not, laboratory personnel should manually dilute the sample and multiply the results by the dilution factor in order to obtain results for the original sample, if applicable to the method. Laboratories should define the maximal permissible sample dilution. Although there are no universal guidelines on maximal permissible sample dilution, our recommendation is that the laboratory define and examine the dilution protocol based on the measurement procedure and define the range of reporting results, including the AMR and maximum dilution that can be used for each test, as well as the method for which dilution is applicable.

Manual dilution must be performed in accordance with manufacturer’s suggestions. If recommended dilutions are not enough to provide results or if there are no recommendations from the manufacturer regarding dilutions, the laboratory must examine the appropriateness of dilution for the intended use of the test results (with appropriate documentation of the dilution method used). It is important to recognize the importance of such results for a patient’s health, and the information must be given to the clinician in order for appropriate and timely action to be taken. If the test result is issued using a dilution that is not recommended, the result should be issued together with a comment on how it was obtained.

#### 1.3.2. Repeat testing

**Recommendation 7**

Repeat testing is recommended only when results are flagged by the analyser, regardless of their position within or beyond the AMR.

Most MBLs repeat a certain percentage of their tests in order to verify their accuracy, even if they previously verified the performance of their analysers. Tests giving results outside the relevant reference intervals are repeated more often than those giving results within reference intervals. Retesting prolongs turnaround time (TAT) and increases laboratory costs. All results flagged by an analyser have to be confirmed by retesting, regardless of their position within or beyond the AMR. Laboratories should establish rules for repeat testing. Regardless of whether retesting is performed, physicians may request new samples if a test result is inconsistent with a patient’s condition or with the previous result (*37*-*39*). Similarly, if the laboratory expert has any doubts about a test result, he or she may also request a new sample. It is recommended that each laboratory set rules for repeating certain results.

#### 1.3.3. Communication with a physician/clinical department about possible causes of unexpected results and/or about the need for new sampling

Sometimes the reason why test results are inconsistent with a patient’s other analyses can be established through communication with the attending physician or clinical department. The reason may be pre-analytical error, interference, or a particular therapy or diagnostic procedure. Such communication can facilitate the definition of further procedures, such as resampling, and guide the decision whether to release the laboratory test report with or without the unexpected test result.

#### 1.3.4. Reflex testing

Reflex testing (protocol testing) is defined as automated addition of tests to be performed depending on the result of primary testing and based on predefined algorithms established by laboratory experts (*40*). For example, a test for direct (conjugated) bilirubin is performed if a test for total bilirubin gives a result higher than the upper limit of the reference interval. As another example, free thyroxine (fT4) is performed if thyroid-stimulating hormone (TSH) results are outside the reference interval. Algorithms to trigger reflex testing are a part of the analytical software programme or LIS. Laboratory experts decide which tests are to be included in these algorithms, based on consultation with physicians and/or accepted clinical guidelines (*40*).

#### 1.3.5. Reflective testing

Reflective testing is a non-automated procedure in which laboratory experts add additional tests and/or comments to the original request, after consideration of a wide range of information, including previously obtained laboratory results, clinical information, and demographic data. Reflex testing and especially reflective testing are considered useful for improving the diagnosis and treatment of patients (*40*, *41*). Before the introduction of reflective and/or reflex testing in routine laboratory work, it is necessary to inform physicians about this possibility. Such testing may form part of the recommendations for further actions in the “Comments” area on laboratory test reports (Procedure 5).

**Recommendation 8**

Reflex testing and especially reflective testing are recommended as a useful way to improve diagnosis and treatment. Before the introduction of reflective and/or reflex testing in routine laboratory work, agreement must be reached within the laboratory and with physicians, as well as in alignment with accepted clinical guidelines, about which tests are to be included in these algorithms.

## PROCEDURE 2: Decision to release test results

After review of the results of tests and any additional procedures, the decision is made whether to release the test results. This decision is made based on all factors that may have influenced the results, including clinical condition and diagnosis, treatment procedures, as well as pre-analytical and analytical factors. If the decision is taken not to release the test results, Procedure 5 (“Reporting of test results”) is applied. The decision not to release test results and the reason(s) for the decision should be communicated to the requesting physician. The requesting physician may request testing of a new sample but cannot cancel the laboratory request. The laboratory report should be released without the incorrect result(s) together with an appropriate comment.

### 2.1. Competences of decision-making laboratory personnel

The Law on Medical Biochemistry Practice in Croatia requires that a MBL assign certain laboratory personnel to systematically assess and confirm the results of laboratory tests. These personnel must hold master’s degrees in medical biochemistry and laboratory medicine for the authorisation of general tests, and they must have completed a specialisation in medical biochemistry and laboratory medicine for the authorisation of highly complex tests, in accordance with the CCMB ordinance on test types (*9*).

These assigned individuals are the only laboratory personnel authorised to access patients’ medical histories, partially or completely revoke laboratory test results, change test results on laboratory reports that have already been issued and modify patients’ personal data. On the basis of clinical information and previous test results, they may request retesting or resampling. They make the final decision about the accuracy of the test results, and they are responsible for confirming the results.

**Recommendation 9**

Only laboratory personnel with master’s degrees and/or with a completed specialisation in medical biochemistry and laboratory medicine have the necessary competencies to confirm test results and decide whether to release them after review and any additional procedures.

## PROCEDURE 3: Preparation of the laboratory test report

**Recommendation 10**

A laboratory test report has to meet the minimum content and layout requirements as shown in Table 2 and detailed in Appendices 1 and 3.

Once it has been decided to release the results of laboratory analysis, the confirmed results are prepared in the form of a laboratory test report. If there is any doubt about any results, the laboratory test report should be released without these results, and relevant information should be provided in the “Comments” area to enable correct clinical interpretation and, if appropriate, future procedures can be recommended. Here, for example, reflex and/or reflective testing may be recommended if this was not already performed during Procedure 1 (“Evaluation of test results”).

### 3.1. Content and layout of the laboratory test report

The most important attributes of the laboratory test report are the use of recommended, standardised language and syntax and the presence of all administrative and patient identification data, measurement results and confirmation data. Where appropriate, the report should also include comments necessary for interpretation of the test results and references and details for highly differentiated laboratory procedures (*16*). Comments are added only to improve the clinical value of the results and influence further diagnostic procedures or differential diagnosis; comments that do not provide additional value to the results should be avoided. Table 2 describes the minimum required content of a laboratory test report (*16*, *42*-*45*).

**Table 2:** Minimum required content of a laboratory test report

**Administrative data**	1. Name, address and telephone number of the medical institution and medical biochemistry laboratory; name, surname and qualification of the laboratory head; name and address of the laboratory location (if distinct from the medical institution) 2. Name of the recipient of the laboratory test report, *i.e.* the person who requested the analysis (name and surname of a physician) 3. A unique patient identifier and the location on the laboratory test report 4. Date and time of sampling 5. Date and time of sample receipt 6. Date and time of laboratory test report release 7. Unique identifier for the laboratory test report and numbering of all pages, together with the total number of pages 8. Name and contact information of the department to which the test results are linked, if the results from multiple laboratory departments are combined onto a single laboratory test report
**Patient identification information**	1. Name and surname 2. Gender 3. Date of birth 4. Unique national health insurance identification number 5. Sample barcode
**Attributes of** **measurand**	1. Sample type 2. Full name of the analyte and/or internationally accepted abbreviations for all tests 3. Appropriate marking of test results outside reference intervals 4. Results should be in SI units, where applicable 5. Defined decimal places for each numerical value, where applicable 6. Reference intervals according to age and gender, where applicable 7. Diagrams/nomograms showing the categories of clinical decision, where applicable (*e.g.* elpherogram) 8. Comments and other remarks
**Confirmation of data**	1. Data of the responsible laboratory expert who authorised the laboratory test report (name, qualifications and medical insurance identification number) 2. Electronic signature of the responsible laboratory expert who authorised the laboratory test report, if possible
**Comments**	1. Comments on sample quality that may have negatively affected the analysis 2. Comments on sample stability and acceptability if sample is not within the laboratory defined criteria 3. Where applicable, comments about analysis results, which may include automatically generated interpretations 4. Name of the person requesting additional tests to be performed 5. Name of the person responsible for continuation of analysis of samples of unacceptable quality 6. Identification of tests that are part of a research or development programme and for which special requests are not required (in the case of laboratory tests made for the purpose of medical research) 7. Patient history of drug treatment and possible interferences

In addition to the information described in Table 2, the laboratory test report should use the terms “reference interval”, “therapeutic interval”, “recommended values” and “cut-off values” in accordance with CCMB guidelines (*16*). The laboratory test report does not need to indicate the names of those who performed the sampling, received the sample in the laboratory or those who performed the analysis. However, this information should be recorded in the LIS. An electronic overview of the laboratory test report should bear one of the following statements (or similar): (a) „This laboratory test report has been printed from the laboratory information system and is legally valid without a stamp or signature.”; (b) “This is a printed copy of a laboratory report that is archived electronically and can be reproduced.”; (c) “This is a printed form of an electronically authorized laboratory report.”; or (d) “This is a laboratory test report printed from the laboratory information system.” It is necessary to indicate the place and time where a printed version of the laboratory report with authorised signature can be obtained. Appendices 1 and 3 show examples of a well-organised laboratory test report and most common standard comments related to pre-analytical, analytical and post-analytical phases of laboratory work.

## PROCEDURE 4: Release of the laboratory test report

**Recommendation 11**

**We recommend electronic release of laboratory test reports whenever possible, but it must always be possible to obtain a printed form. Electronically released laboratory test reports must be in a “read-only” format that permits no alterations, and measures should be in place to ensure that information is transferred only to authorised computers or printers.**

**If releasing laboratory test reports includes sending them electronically to the patient or the requesting physician via e-mail, the laboratory must receive signed consent from the patient or physician.**

**Results released orally must be supported later with an electronic or printed laboratory test report. The laboratory must document results that are released orally.**

**The laboratory should record policies and procedures about releasing reports, including details about who releases reports and to whom. A laboratory test report can be revoked or changed at any time for objective reasons that must be documented and archived.**

The laboratory test report can be released in electronic and/or printed form. In a LIS connected to a hospital information system (HIS), laboratory test reports that have been confirmed can be printed out or sent electronically, for example, to the e-mail address of the patient or requesting physician. Before sending an e-mail, a patient must be informed that delivering the laboratory test report by e-mail is an unprotected way of sending the data and that the laboratory test report will be sent only with the patient’s signed consent. By making such a statement, the patient accepts the risk of sending the laboratory test report by e-mail. Each laboratory should determine which release channels will be used. In any case, it must always be possible for reports to be printed out and, for example, supplied to the patient upon request or mailed to his or her home address.

Those requesting tests from a laboratory should be informed in advance about how laboratory test reports are released and what the responsibilities of the personnel involved in this process are. The laboratory should record policies and procedures about releasing reports, including details about who releases reports and to whom (*7*). If a physician requests the release of results for only a subset of requested tests, the laboratory test report should be considered incomplete and provisional. The final laboratory test report must be released when all requested tests are completed.

The MBL should specify how printed reports are delivered, distributed and disposed of. These procedures should ensure the protection of the reports themselves as well as the privacy of patient data. When reports are released electronically, they should be in a “read-only” format that permits no alterations, and measures should be in place to ensure that information is transferred only to authorised computers or printers (*7*).

It must be possible to revoke a laboratory test report at any time for objective reasons, such as a change in data about the patient or the requesting physician or department. A laboratory expert is authorised to revoke an entire laboratory test report, part of the report or only a specific test result on the report. Reasons for the revocation should be explained to the medical professional who received the report and used it for decision-making. The laboratory is obliged to archive revoked or modified laboratory test reports (*46*).

When a laboratory test report must be changed, the change should be clearly indicated and explained to the user. The modified report should indicate the date and time of the change, as well as the name of the person responsible for the change. Original results that are later revised should be retained on a cumulative laboratory test report and must be clearly labelled as such. Additions or modifications must be documented, even if the LIS lacks that capability. The laboratory should have a documented plan for unexpected failure of the LIS. In the case of prolonged failure, the laboratory should consider reporting results as original records from the analyser or transcripts on the previously prepared laboratory forms. Results released in this form should be considered as provisional. Results released orally must be supported later with an electronic or printed laboratory test report. Laboratories must document results that are released orally (*46*, *47*).

Effective post-analytical work in referral laboratories requires timely communication between the requesting (referring) laboratory, which organised the sending of the sample, and the referral laboratory, where the tests were performed. The interaction between these laboratories should be specified in a contract, including the methods for reporting and transferring results (i.e. original results, e-mail, fax) and for reporting critical results. The contracted laboratory retains original reports and releases copies to the requesting laboratory. The requesting laboratory should specify any additional conditions when it sends the sample. Additional guidelines defining the interaction between the two laboratories can be found in the CCMB Recommendations for Sampling in a Collaborative (Referral) Laboratory (*48*).

## PROCEDURES 1-4: Automated selection and reporting of test results

**Recommendation 12**

Automated or semi-automated selection and reporting of test results is the recommended procedure. Unambiguous and clearly defined criteria and rules must be assured in order to prevent the release of incorrect laboratory reports. Rules should be set according to laboratory operating procedures. All rules in an automated selection algorithm are equally valuable, and all test results need to be checked against all rules, as described in Appendix 4.

Informatization is an essential part of laboratory work. It has long played a role in pre-analytical and analytical phases of laboratory work, and its role in the post-analytical phase has been increasing, especially in the automatic selection and reporting of test results (Procedures 1-4).

The most useful definition of automated selection and reporting of test results is the process of selecting, confirming and releasing laboratory test reports using software (*49*-*53*). Several types of software can accommodate automated selection and reporting results in routines:

- an independent programme (*54*-*57*),
- LIS after appropriate upgrade (*49*, *51*, *52*), and
- middleware (*53*, *58*).

Before automated selection and reporting test results can be introduced in to a laboratory’s workflow, the laboratory must decide whether it will be semi-automated or automated. When semi-automated selection and reporting of test results is established, laboratory staff must initiate it by selecting the „automated selection” function and enabling the confirmation and release of test results as long as results are available for all tests on the sample. When automated selection and reporting of test results is established, test results are confirmed and released immediately after the analysis is finished. In addition, the laboratory should decide whether automated selection and reporting test results will be performed at the sample level, in which case results are confirmed and released only when results for all tests on the sample are available; or at the test level, in which case automated selection and reporting test results is performed in “real time”, immediately after a test result is released by the analyser to the LIS. The benefit of using semi-automated selection and reporting of test results is the possibility of controlling this process, such as when a laboratory creates a new algorithm in the LIS. It is technically possible to turn every semi-automated selection into real-time automated selection, after automated selection and reporting of test results becomes part of routine laboratory work. All test results for each sample are checked for all the rules on the test level in automated selection algorithm, but the process of automated selection and reporting of test results can be performed at the sample level or at the test level (“real time”).

Rules for automated selection and reporting results should be set according to laboratory operating procedures because such automation is used only in the post-analytical phase. Automated selection and reporting of test results does not affect routine laboratory work, nor does it affect confirmation criteria for steps preceding automated selection. The rules in an automated selection algorithm must be unambiguous and clearly defined in order to prevent the release of incorrect laboratory reports. All rules in an automated selection algorithm are equally valuable, and all test results are checked against all rules (Appendix 4). While these rules vary across laboratories, they often take into account the following:

- Criteria based on the AMR are usually defined according to criteria that have been established by the reagent manufacturer or obtained in the laboratory during its method validation. Results outside the AMR cannot be confirmed or released during automated selection and reporting of test results (*53*).
- Pre-analytical and analytical flags raised by the analyser. These flags may occur because of insufficient sample volume, bubbles in the sample, or technical faults during analysis. If results for a test have been flagged by the analyser, that test (or sample) will not be confirmed or released during automated selection and reporting of test results (*50*, *53*, *54*).
- Interference indices (haemolysis, icterus and lipemia) (*59*). If results for a test have been flagged for interference that test (or sample) will not be confirmed or released during automated selection and reporting of test results.
- Delta check. Each laboratory must define delta check criteria based on its patient population. Criteria can vary, but the one most often used is the RCV (see sections 1.2 and 1.2.1). Besides using RCV to define delta check criteria, the maximum permissible time between two measurements should be specified. This interval can be set for each test individually (*i.e.* glucose, creatinine) or for a class of tests (*i.e.* coagulation tests). If two results were determined after an interval exceeding the maximum, the test result will not be confirmed or released during automated selection and reporting of test results (*25*, *53*-*55*, *57*, *60*).
- Critical results, which are defined by each laboratory. Any result defined as a critical result cannot be confirmed or released during automated selection and reporting of test results (*46*, *47*). Appendices 5 and 5.1 provide critical limits recommended by the CCMB (*61*).

Rules in the algorithm for automated selection and reporting of test results may compare results between different but related tests. For example, test results will not be confirmed or release during automated selection and reporting of test results if the albumin concentration is higher than the total protein concentration. Rules may also be based on reagent lot checks and reference intervals (*50*, *51*, *53*, *55*, *56*). Real-time automated selection and reporting of test results has additional requirements in terms of quality control: if the results of internal quality control fall outside the pre-specified performance criteria, automated selection and reporting of test results will be automatically deactivated until internal quality control results fulfil the specified criteria of acceptance.

Before rules for automated selection and reporting of test results can be implemented in routine laboratory practice, they should be validated for the laboratory’s patient population (*49*, *53*, *58*). During this validation, reports released during automated selection and reporting of test results are expressed as percentages of the values obtained manually by a laboratory expert. Such validation can help guide improvement of the initial algorithm for automated selection and reporting of test results. This validation process should be documented in detail in order to satisfy the requirements for releasing laboratory results. Every error in the algorithm should be tested before introducing automated selection and reporting of test results into the routine. Since the number of samples included in the validation process is not predetermined, each laboratory should decide how many samples will be included in the validation of the algorithm for automated selection and reporting of test results. It is necessary to document all that is done during validation (*e.g.* the number of samples included in validation, which rule most often stopped automated selection and reporting of results, how many samples were not confirmed or not released by automated selection and reporting of test results as well as for which reason).

Automated selection and reporting of test results simplifies post-analytical laboratory work, shortens TAT, and helps ensure that results are confirmed based on the same objective rules, without risk of inter-individual variation such as among laboratory personnel. Automated selection and reporting of test results can reduce the number of test results that require manual checking, allowing laboratories to focus on potentially problematic samples. Nevertheless, automated selection and reporting of test results can not completely replace the work of laboratory experts, who are critical in interpreting the reasons why a particular result could not be confirmed and released by the process of automated selection and reporting of test results. The process of automated selection and reporting of test results must have a “stop button” in order to prevent release of an erroneous laboratory test report.

## PROCEDURE 5: Reporting of test results

Each laboratory must define how it reports test results, and this reporting must be accurate, clear and unambiguous. If results are communicated by telephone, the content of the communication, the authorisation to give and receive the information, as well as the manner in which the communication is recorded and stored in laboratory documentation must be defined. Any verbal communication of test results must be considered provisional and must be followed up with a written or electronic report.

Particularly important are the results of critical laboratory tests that require immediate medical attention (*46*). Depending on user needs, the laboratory can define critical tests requiring timely reporting because they have immediate influence on patient care.

Furthermore, some results may be described as significant-risk results indicating the risk of important adverse outcomes and therefore requiring medical attention in a clinically justified time frame (*62*, *63*). Each laboratory should compile a list of laboratory tests for which critical limits should be defined, and these limits should be established in consultation with the physicians who use the laboratory’s services and following the CCMB recommendations on “Critical Laboratory Findings and Critical Result Reporting” (Appendix 5) (*61*). Laboratories are encouraged to verify the critical limits and procedure for reporting critical results in order to reach consensus on effective and appropriate communication of critical results within the clinical community they serve (*64*). The laboratory can choose to define critical limits separately for in- and outpatients, depending on physicians’ needs, specificities of the patient population, extent of laboratory services and type of health care provided at the medical institution. Critical limits may even be defined for specific departments or clinical units. The International Federation of Clinical Chemistry (IFCC) includes a laboratory’s reporting of critical results among its quality control indicators of the post-analytical phase (*65*, *66*). The effective way of configuring and further monitoring should be chosen by each laboratory depending of the LIS capabilities and clinical environment, with due recognition of the limitations of each step in reporting critical results, such as the number of results to report, the ward and/or clinician(s) involved, and available communication channels.

**Recommendation 13**

Each laboratory should compile a list of laboratory tests for which critical limits should be defined. Critical limits of laboratory results should be established in consultation with the physicians who use the laboratory’s services and following the CCMB recommendations as presented in Appendix 5.

### 5.1. Reporting of critical results

Once a laboratory has established critical limits of laboratory test results, it should define the procedures for reporting critical results to physicians or other authorised medical personnel. This definition process includes specifying how results are reported, within what time frame they must be reported, and which laboratory personnel are responsible for reporting them. Critical results should typically be reported within 30 minutes of confirmation; waiting for re-testing can delay reporting unnecessarily because it increases the reliability and safety of results only slightly (*38*, *46*, *62*, *67*, *68*). Only authorised personnel can report critical results. All test results must be confirmed by a qualified member of laboratory personnel with a master’s degree in medical biochemistry and laboratory medicine or a specialisation in medical biochemistry and laboratory medicine.

Critical reporting can be done verbally, and all reported results must be read-back by the receiver of the information in order to avoid misunderstanding. In addition, the verbal communication of critical results must always be followed by a written or electronic report. Limits for critical results can also be included in the LIS, facilitating their rapid interpretation. If a laboratory test report is released electronically, channels must be provided for immediate delivery and receipt confirmation. The channels can include intercom, e-mail, fax, or other forms of communication that allow the information to be given to a predefined person within a predefined time. Key points of critical result reporting are present in Recommendation 14.

**Recommendation 14**

Critical results have to be reported within 30 minutes of confirmation; waiting for re-testing can unnecessarily delay reporting.

A report of a critical result must contain at least the following:

- Name and surname of the patient, name of the department and laboratory identification number;
- Critical result;
- Name and surname of the person reporting the critical result;
- Method or channel of critical result reporting (if multiple channels are used);
- Time of report;
- Name and surname of the physician or other authorised medical personnel receiving the notification.

Only authorised personnel can report critical results. All test results must be confirmed by a qualified member of laboratory personnel with a master’s degree in medical biochemistry and laboratory medicine or a specialisation in medical biochemistry and laboratory medicine.

## PROCEDURE 6: Sample storage and disposal

**Recommendation 15**

Minimum sample storage conditions for traceability purposes are presented in Appendix 6.

The laboratory must have a documented procedure for identifying, collecting, marking, accessing, storing and safely disposing of biological samples.

Primary samples must be stored after analyses to ensure their availability for re-testing or additional testing. Laboratory personnel must be well trained about whether a certain test can be repeated or performed for the first time on a stored sample. Operating procedures for each analyte should stipulate acceptable storage conditions and duration. The laboratory must have a documented procedure for identifying, collecting, marking, accessing, storing and safely disposing of biological samples. The laboratory must define durations of storage for biological samples (*69*-*72*).

Optimal storage conditions and duration depend on the type of sample, analyte stability and analyte half-life and the type of test being carried out (*7*). Generally, serum or plasma can be stored for 4 hours at room temperature in primary uncapped tubes, 48 hours at 4 ºC in primary capped tubes, and several days to several months at - 20 ºC in secondary capped tubes. The storage conditions and longest storage time of samples should be noted if re-tests or additional tests are needed from the stored sample. When a requesting laboratory sends a sample to a referral laboratory, the shipment should be documented, and an aliquot of the sample should first be removed and stored at - 20 ºC in dedicated freezer space until results are received (*69*). Appropriate measures must be undertaken to prevent sample contamination and degradation. Appendix 6 describes minimum sample storage conditions for traceability purposes (*69*-*72*).

Temperature should be controlled in the same way for refrigerators carrying already analysed samples and for refrigerators storing samples before testing, reagents, calibration standards and control samples. Temperature control should be regularly monitored automatically/electronically or manually by checking an appropriately positioned thermometer which should be regularly calibrated in a traceable way (*7*). If an institution archives samples for education, research, or other public health interests, it should define the conditions and duration of their storage. Laboratory experts can decide to prolong the archiving of results and materials for the purposes of laboratory monitoring, education, epidemiology monitoring, or statistical studies.

Samples must be disposed safely in accordance with local regulations and recommendations. In Croatia these are contained within the Law on Sustainable Waste Management and its amendments, Ordinance on Waste Types, Ordinance on Management of Medical Waste, Regulations on Categories, Types and Classification of Waste with Waste Catalogue and List of Hazardous Waste, and the Croatian Ministry of Health Recommendations on Treatment of Waste Resulting from the Provision of Health Care (*73*-*79*). Laboratories and their home medical institutions may also define additional regulations.

## PROCEDURE 7: Archiving of laboratory documentation

**Recommendation 16**

Minimum archiving conditions of laboratory documentation according to CCMB recommendations is described in Appendix 7.

Recording and maintenance of medical documentation is a general (public) duty of health care professionals and health care institutions and is governed by various laws. The daily processes in a laboratory generate substantial amounts of data, mostly in electronic form, that must be catalogued and archived to ensure credibility and quality of test results. Laboratory documentation must be archived efficiently to save money and space, improve productivity, allow rapid information sharing, protect patient privacy and be environmentally sustainable (*70*-*72*).

In most cases, patient medical records are stored in the HIS, and these data are merged into a single electronic health card (EHC). In an MBL, however, data are usually stored on a computer or shared storage platform (server) within the laboratory. Archiving of laboratory documentation means storing all important and meaningful data and notifications in a format that is dated and certified and can protect the data for a minimum period of time (*7*). These minimum storage periods vary with the type of document.

Appendix 7 describes CCMB recommendations on minimum archiving conditions for laboratory documentation (*80*). When necessary, these requirements can be adapted to the requirements of local health care institutions. Croatian and European Union (EU) legislation permit documentation to be archived in paper or electronic form. In any case, the archiving system must protect against documentation loss or damage through fire, water, environmental conditions, insects, rodents, microorganisms, theft and accidents. Special measures are needed to protect electronic patient data from abuse, mostly in the form of unauthorised use by others. Therefore, laboratories must define carefully who can access patient data for what purpose, and the data must be protected from alteration, premature destruction or unauthorised use (*81*).

If the LIS is linked to the HIS, each employee using the HIS system should be assigned an account and should be required to log out after each use, in order to prevent others from taking advantage of that employee’s access privileges. Employees are required to maintain the confidentiality of this information. The EU has recently released extensive requirements and guidelines for protecting personal data in its General Data Protection Regulation (GDPR) (*82*). One of the basic tasks that GDPR requires of organisations is to protect the personal data of their customers/users. Organisations must at all times know where and for what purpose information may be used. In the event that someone decides to withdraw consent to the use of their personal data, the organisation must be able to honour that within the prescribed deadline. The importance of personal data in the medical biochemistry system applies primarily to the name, address, email address, IP address and access point (MAC), global positioning system (GPS) location, telephone number, video recordings of individuals, identification number, biometric data (genetic data, educational and professional information, health data, sexual orientation) and other data relating to an individual whose identity is identified or can be identified. This European regulation will not affect all activities in Croatia equally, but it will certainly have a significant impact on the health care system. Health data are classified as particularly sensitive data. For this reason, institutions and companies that process health data will be under the special care of the agency responsible for the protection of personal data in the Republic of Croatia (*82*).

Only authorised personnel during the normal exercise of their duties should have access to patient medical data. If necessary, HIS administrators can determine the identity of users who enter or alter records because the HIS activity log associates such changes with the account of the particular user.

Laboratories should define who is responsible for requesting accounts for personnel. Once the account is created, each employee is required to change the initial password through the programme module. Medical documentation in the HIS is informational: only printed documentation that has been verified and signed by an appropriate individual has legal standing (*82*).

## PROCEDURE 8: Post-analytical quality indicators

**Recommendation 17**

Monitoring of quality indicators in daily laboratory work is recommended.

Minimum recommended quality indicators for the post-analytical phase are: turnaround time (TAT), percentage of incorrect (revoked) laboratory test reports, and notification of critical results.

Monitoring quality indicators in daily work can reduce laboratory errors and risk to patient safety by identifying problems in all phases of laboratory process, allowing their correction (*66*, *83*). *Per* the ISO 15189:2012 requirements 4.14. “Evaluation and audit” and 4.14.7 “Quality indicators”, each laboratory should define quality indicators for the pre-analytical, analytical and post-analytical phases of laboratory work and how the phases will be monitored. Quality indicators must be clearly and unambiguously defined and capable of being monitored (*65*, *84*). Each laboratory decides on its own which quality indicators will be implemented in each phase of the laboratory work (*7*). Minimum recommended quality indicators for the post-analytical phase are discussed below (*85*).

### 8.1. Turnaround time

**Recommendation 18**

Turnaround time (TAT) is defined as the time interval that starts from the time when the laboratory receives the sample until the time the test results for that sample are validated and released (*86*).

Monitoring of turnaround time can be expressed in terms of percentage of tests not performed within a given time (*86*). Each laboratory should define turnaround time for each test according to clinical needs, it should define how turnaround time will be monitored, and it should periodically analyse turnaround times (*7*).

### 8.2. Errors during transcription of results/incorrect laboratory reports

**Recommendation 19**

Monitoring and periodical analyses of all laboratory test reports incorrectly released for any reason, as well as monitoring the reasons for corrections of laboratory test report are recommended.

The percentage all laboratory test reports that are incorrect is another essential quality indicator in the post-analytical phase. It may be useful for detecting the most frequent causes of erroneous reports (*83*). It can be calculated in terms of the percentage of manually transcribed results that were incorrectly transcribed, in terms of the percentage of results released by the LIS that were incorrect, or in terms of the percentage of released reports that were incorrectly released (*5*). All corrections in laboratory test reports should be documented; preserving evidence about the initial results. The reasons for corrections should also be documented on the reports, as well as the name of the person by whom the correction was made.

### 8.3. Notification of critical results

**Recommendation 20**

All procedures related to the reporting of critical results have to be recorded and the data periodically analysed (number or proportion of critical results reported within a defined period of time).

Sections 5 and 5.1 describe the importance of defining critical limits of laboratory test results for crucial medical tests. Quality control can be assessed in terms of how quickly these values are reported to appropriate medical personnel. This indicator can be expressed as the average time required for reporting, or as the number or proportion of critical results reported within a defined period of time (*84*). All procedures related to the reporting of critical results should be recorded and the data periodically analysed. The ISO 15189:2012 requirement 5.9. “Release of results” demands that actions in response to critical results be recorded. Furthermore, the IFCC position paper on quality indicators states that this indicator should be monitored (*66*). Each laboratory should choose an effective way to configure and further monitor procedures for reporting critical values, in accordance with the LIS capabilities and clinical environment and in recognition of the limitations of each step in the reporting of critical results, such as the number of results to report, the ward and/or physician(s) involved, and the available communication channels.

## Conclusion

The Law on Medical Biochemistry Practice and quality control principles dictate that every laboratory define staff who systematically assess and validate the results of laboratory testing. Every laboratory must implement procedures that ensure that test results are evaluated, before release, by authorised personnel on the basis of available clinical information and previous test results, and that test results are evaluated for quality control purposes. Decisions about the acceptability of laboratory test results should be based on the patient’s diagnosis, the laboratory tests available, how the sample was collected and stored, the presence of potential interferences, and the reliability of analyser measurements based on internal and external quality controls and limit values determined based on the laboratory’s target population. All actions, including additional procedures, must be documented and analysed in an effort to reduce the need for repeat blood sampling and re-testing.

Each laboratory determines the authority and degree of responsibility of each member of personnel in accordance with that person’s documented competencies. It also defines the responsibilities and authority shared among the entire staff, such as searches and release of test results as well as communications of results by telephone. The responsibilities of laboratory personnel need to be aligned with the Law on Medical Biochemistry Practice and CCMB documents; they should be recorded and explained to the laboratory personnel.

Laboratories should conform to recommendations of the CCMB and CSMBLM and to internal policies and regulations of the healthcare facilities where the laboratories operate. This includes recommendations about general and specialised skills as well as competencies and qualifications required for laboratory personnel engaging in each type of work.

Test results must be reported accurately, clearly and unambiguously in a manner consistent with the specific instructions in the test operating procedures. Every laboratory must define the format of laboratory results, whether electronic or paper-based, and the manner in which they are released from the laboratory. These policies must take into account the recommendations of professional organisations, as well as the needs and demands of physicians and patients.

## APPENDIX 1 Example of a laboratory test report

1. Obligatory administrative information about the institution: name, address and telephone number of the medical institution and medical biochemistry laboratory, name and surname of the head of laboratory and his or her qualifications, name and address of the laboratory (if different from the hospital), electronic mail and websites of institutions and laboratories.

2. Obligatory administrative data on the patient, name of the recipient of the laboratory results or the person who requested the test (name and surname of the physician), unique identifier of the patient’s location and destination of the report, ambulance, clinic or departmental mark, date and time of sampling, date and time of receipt of the sample and date and time of release of the laboratory test report. If data are electronically recorded in the LIS, data on the individuals who performed the sampling and who received the sample need not be displayed here. Also, the name of the laboratory personnel who performed analysis is not obligatory. These data can be stored in the LIS.

3. Information about the laboratory unit that generated the indicated results, data on the manager of the department or laboratory unit, his or her qualifications, telephone number, and electronic mail. The testing system and type of primary sample should be described using abbreviations defined in the report. The type of analyte should be indicated using the full name from the guidelines in “Harmonisation of Laboratory Reports in the General, Specialist and Highly Differential Medical Biochemistry” and/or internationally accepted abbreviations for all tests (*16*). Exceptions are components of complete blood count and acid-base balance values. The meaning of annotations with asterisks, H and L or bold facing for values outside the reference interval are clarified here. Test results should be presented in SI units and expressed in numerical values to a defined number of decimal places according to harmonisation of laboratory results or qualitative values. The reference interval corresponds to the patient’s age and sex and, preferably, to the population to which the patient belongs. Recommended terms are “reference interval”, “therapeutic range”, “recommended values”, and “limit (‘cut off’) values” according to the guidelines in the document “Harmonisation of Laboratory Results in the Field of General, Specialist and Highly Differentiated Medical Biochemistry” (*16*). In this part of the report, diagrams or nomograms can be added to support clinical decision-making. In the “Remarks” column of part 3, the appropriate reference interval and diagnostic values are indicated together with their sources, which should be consistent with the recommendations of CCMB and CSMBLM. This is particularly important for the oral glucose tolerance test in pregnancy, glomerular filtration rate (eGFR-CKD-EPI), albumin and creatinine ratio in urine, protein ratio and urine creatinine (*87*, *88*).

4. Comments clarify the essential parts of the report or the test procedures performed, *e.g.* they highlight the presence of critical results on the laboratory test report or provide treatment information. If no reference interval is available, the reference interval should be clearly indicated. If the reference interval of laboratory test results is recommended by the national harmonisation documents, it is sufficient to indicate the source of the applied reference interval. In situations where the reference interval comes not from national harmonisation documents but from another source, this source should be stated. If a reference interval is completely absent, this should be indicated in the “Comments” area. Comments may relate to sample quality in terms of acceptance and rejection criteria, the most common interferences potentially affecting the quality of the results, reasons for non-disclosure of the report, together with the surname and name of the individual requesting additional tests. Data are provided about the person who assumed responsibility for continuing the test with a sample of unacceptable quality. Where appropriate, test results can be interpreted based on analyses from molecular diagnostics and genetic testing, analytical toxicology, and immunology. In conclusion, this is a space for writing all the information that may be useful when making a medical decision.

5. Mandatory notes to be found on each laboratory test report include type of laboratory test reports and an indication of when and where it is possible to obtain authorised printed versions of the report. In the “Flag” column, abbreviations for values above the upper and below the lower limit of the reference interval should be defined. Also, it should be indicated that all tests were performed by the recommended methods and have recommended reference intervals from the mandatory national harmonisation document (*16*). Otherwise, the method and the origin of the reference intervals need to be mentioned in the “Comments” area. If a report contains the results of immunochemical methods, the following sentence must be added: „Test results obtained with different immunochemical methods cannot be compared“.

6. The legend of sample types is adapted to the type(s) of sample actually used.

7. The page designation also indicates the total number of pages.

8. If the report is released in original form as an original report, it is necessary to state the data and signature of the authorized person and verified with a stamp. If the report is released electronically, the signature of the approving individual is not required. The original form of the report is printed, manually signed and stamped, and released in the MBL; only in this case is the report legally valid.

## APPENDIX 2 Example of delta check calculations for a laboratory test of creatinine

**Table:**

**Delta check calculation**		
Result	Current result	Creatinine = 79 µmol/L
	Previous result	Creatinine = 42 µmol/L
Delta difference	37 µmol/L	
Delta percent change	88.1%	
Calculation of delta check limits based on analytical variability and intra-individual biological variability		
Analytical variability, CV_A_*	1.60%	
Within-subject biological variation, CV_I_	5.95%	
RCV (95% confidence interval)	2^1/2^ x 1.96 x (1.6^2^+5.95^2^)^1/2^	
	= 17.0%	
Assessment of results based on delta check limits		
Delta check limit	< 17.0%	
Conclusion	The result is outside the defined delta check limits	

***CV_A_ from long-term internal quality control data

## APPENDIX 3 Most common standard comments for different situations during the pre-analytical, analytical and post-analytical phases of laboratory work

**Table:**

**Comments referring to situations during the pre-analytical phase**		
**Comment**	**Location on report**	**Authorised personnel**
Blood sample (analyte/group of analytes) collected in wrong container. Please recollect the sample in tube with/without anticoagulant.	With the analyte result Under the group of analytes At the end of the report	Medical laboratory technician Bachelor of medical laboratory diagnostics Master of medical biochemistry and laboratory medicine Specialist of medical biochemistry and laboratory medicine
Insufficient sample volume (analyte/group of analytes). Please recollect the sample to the fill mark on tube.	Under the group of analytes At the end of the report	Medical laboratory technician Bachelor of medical laboratory diagnostics Master of medical biochemistry and laboratory medicine Specialist of medical biochemistry and laboratory medicine
Insufficient volume of submitted urine/stool sample for testing of (analyte/group of analytes).	Under the group of analytes At the end of the report	Medical laboratory technician Bachelor of medical laboratory diagnostics Master of medical biochemistry and laboratory medicine Specialist of medical biochemistry and laboratory medicine
Blood/urine sample unlabelled. Please recollect with appropriate sample identification.	With the analyte result Under the group of analytes	Medical laboratory technician Bachelor of medical laboratory diagnostics Master of medical biochemistry and laboratory medicine Specialist of medical biochemistry and laboratory medicine
Blood sample clotted. Please recollect with appropriate sample mixing.	With the analyte result Under the group of analytes	Medical laboratory technician Bachelor of medical laboratory diagnostics Master of medical biochemistry and laboratory medicine Specialist of medical biochemistry and laboratory medicine
Blood sample not submitted to the laboratory within defined time. Please recollect the sample.	With the analyte result Under the group of analytes At the end of the report	Medical laboratory technician Bachelor of medical laboratory diagnostics Master of medical biochemistry and laboratory medicine Specialist of medical biochemistry and laboratory medicine
Blood/urine sample not submitted to laboratory at all.	With the analyte result Under the group of analytes At the end of the report	Medical laboratory technician Bachelor of medical laboratory diagnostics Master of medical biochemistry and laboratory medicine Specialist of medical biochemistry and laboratory medicine
Patient not properly prepared for sample collection.	With the analyte result Under the group of analytes At the end of the report	Medical laboratory technician Bachelor of medical laboratory diagnostics Master of medical biochemistry and laboratory medicine Specialist of medical biochemistry and laboratory medicine
**Comment**	**Location on report**	**Authorised personnel**
Inappropriate collection of 24-hour urine. Please recollect following attached instructions.	With the analyte result Under the group of analytes	Medical laboratory technician Bachelor of medical laboratory diagnostics Master of medical biochemistry and laboratory medicine Specialist of medical biochemistry and laboratory medicine
If the patient is on oral iron therapy, the sample should be recollected after proper preparation.	With the analyte result	Medical laboratory technician Bachelor of medical laboratory diagnostics Master of medical biochemistry and laboratory medicine Specialist of medical biochemistry and laboratory medicine
Plasma/serum haemolysed. Please recollect the sample for (analyte/group analyte).	With the analyte result Under the group of analytes	Medical laboratory technician Bachelor of medical laboratory diagnostics Master of medical biochemistry and laboratory medicine Specialist of medical biochemistry and laboratory medicine
Plasma/serum lipaemic/icteric. Because of interference, it was not possible to measure (analyte/group analyte).	With the analyte result Under the group of analytes	Master of medical biochemistry and laboratory medicine Specialist of medical biochemistry and laboratory medicine
**Comments referring to situations during the analytical phase**		
Platelet count from sample collected in sodium-citrate/ lithium-heparin/EDTA tube.	With the analyte result Under the group of analytes	Medical laboratory technician Bachelor of medical laboratory diagnostics Master of medical biochemistry and laboratory medicine Specialist of medical biochemistry and laboratory medicine
Results measured in capillary blood.	Under the group of analytes	Medical laboratory technician Bachelor of medical laboratory diagnostics Master of medical biochemistry and laboratory medicine Specialist of medical biochemistry and laboratory medicine
Pre/post-haemodialysis. Pre/post-filter. With/without oxygen (comments entered by nurses/physician).	With the analyte result Under the group of analytes	Medical laboratory technician Bachelor of medical laboratory diagnostics Master of medical biochemistry and laboratory medicine Specialist of medical biochemistry and laboratory medicine
Recollected sample.	Under the group of analytes	Medical laboratory technician Bachelor of medical laboratory diagnostics Master of medical biochemistry and laboratory medicine Specialist of medical biochemistry and laboratory medicine
Platelet count confirmed microscopically.	With the analyte result Under the group of analytes	Medical laboratory technician Bachelor of medical laboratory diagnostics Master of medical biochemistry and laboratory medicine Specialist of medical biochemistry and laboratory medicine
**Comment**	**Location on report**	**Authorised personnel**
Differential blood count confirmed microscopically.	Under the group of analytes	Medical laboratory technician Bachelor of medical laboratory diagnostics Master of medical biochemistry and laboratory medicine Specialist of medical biochemistry and laboratory medicine
Because the sample was lipaemic, corrected values for haemoglobin and red blood cell indices are reported.	With the analyte result Under the group of analytes	Medical laboratory technician Bachelor of medical laboratory diagnostics Master of medical biochemistry and laboratory medicine Specialist of medical biochemistry and laboratory medicine
Results obtained by various immunochemical methods cannot be compared	Under the group of analytes At the end of the report	Medical laboratory technician Bachelor of medical laboratory diagnostics Master of medical biochemistry and laboratory medicine Specialist of medical biochemistry and laboratory medicine
Method of determination (XXY), the manufacturer, the analyser	With the analyte result	Medical laboratory technician Bachelor of medical laboratory diagnostics Master of medical biochemistry and laboratory medicine Specialist of medical biochemistry and laboratory medicine
**Comments referring to situations in the post-analytical phase**		
At the physician’s request, results are reported for an analytically inappropriate sample.	With the analyte result	Master of medical biochemistry and laboratory medicine Specialist of medical biochemistry and laboratory medicine
The following tests were made (analyte) upon additional request by the physician.	With the analyte result	Master of medical biochemistry and laboratory medicine Specialist of medical biochemistry and laboratory medicine
Report copy released (date).	At the end of the report	Master of medical biochemistry and laboratory medicine Specialist of medical biochemistry and laboratory medicine
Pseudothrombocytopenia. Please recollect the sample in sodium-citrate tube.	Under the group of analytes	Master of medical biochemistry and laboratory medicine Specialist of medical biochemistry and laboratory medicine
All results of a screening test for drug abuse are not valid without confirmatory measurement.	Under the group of analytes	Medical laboratory technician Bachelor of medical laboratory diagnostics Master of medical biochemistry and laboratory medicine Specialist of medical biochemistry and laboratory medicine
Result revised Report revised. Date (dd/mm/yy). Responsible person (name). Date of original report (dd/mm/yy)	With the revised analyte result At the end of the report	Master of medical biochemistry and laboratory medicine Specialist of medical biochemistry and laboratory medicine

## APPENDIX 4 Example of an algorithm for automated selection and reporting of test results for a serum glucose test

**Example 1.** Glucose concentration, 4.6 mmol/L

Delta check: 15%

Serum indices: haemolysis 2, icterus 0, lipemia 0

**Example 2.** Glucose concentration, 28.0 mmol/L

Delta check: 20%

Serum indices: haemolysis 10, icterus 0, lipemia 0

**Example 3.** Glucose concentration, 5.0 mmol/L

Delta check: 70%

Serum indices: haemolysis 70, icterus 0, lipemia 0

**Table:**

**Rules for automated selection and reporting of test results**		**Specific criteria**	**Example 1**	**Example 2**	**Example 3**
Analytical measurement range		0.1-41.6 mmol/L	YES	YES	YES
Critical values		< 2.5 mmol/L > 27.8 mmol/L	YES	NO	YES
Delta check % (time/days)		Up to 60% / 7 days	YES	YES	NO
Serum indices^*^					
	Lipemia (mg/dL of Intralipid)	< 1000	YES	YES	YES
	Haemolysis (mg/dL of haemoglobin)	< 60	YES	YES	NO
	Icterus (mg/dL of bilirubin)	< 1000	YES	YES	YES
Confirmation and release of test results by automated selection process			YES	NO	NO

^*^Example of reporting serum indices on the Cobas c501 (F. Hoffman-La Roche Ltd).

## APPENDIX 5 Critical limits of laboratory tests (*61*)

**Table:**

**Parameter**	**Value**	**Comment**
**Haematology**		
Haematocrit	< 0.180 (L/L)	Agrees with haemoglobin concentration < 60 g/L. Myocardial oxygen supply inadequate
	> 0.610 (L/L)	Hyper-viscosity of blood, high resistance in blood circulation, high risk of heart failure
Haemoglobin	< 66 g/L	Myocardial oxygen supply is inadequate
	> 199 g/L	Agrees with haematocrit of 0.610 - hyper-viscosity syndrome
White blood cell count	< 2 x 10^9^/L	High risk of infection if the number of granulocytes is 0.5 x 10^9^/L
	> 38 x 10^9^/L	Leukaemoid reaction, *e.g.* in sepsis or leukaemia
Platelet count	< 20 x 10^9^/L	Risk of bleeding. Exclude pseudothrombocytopenia caused by EDTA anticoagulation
	>1000 x10^9^/L	Risk of thrombosis
**Coagulation**		
Activated partial thromboplastin time	75 s	Lack or inactivity of factors VIII, IX or XII, with risk of bleeding
Antithrombin	< 50%	Major lack of inhibitors in patients with a higher procoagulation activity is associated with higher risk of thromboembolic complications
Fibrinogen	< 0.8 g/L	Risk of bleeding
Prothrombin time	< 0.15 (> 40 s) INR ≥ 5	Decrease in factor V and vitamin K-dependent factors II, VII and X. Interference in their synthesis. Risk of bleeding for patients on coumarin therapy
**Biochemistry**		
Serum amylase	> 350 U/L	Pancreatitis or salivary gland infection
Aminotransferases	> 1000 U/L	Values > 500 U/L ALT and > 750 U/L AST can be applied depending on the patient population
Ammonia	> 59 µmol/L	Risk of hepatic encephalopathy
Anion difference	> 20 mmol/L	Ketoacidosis or lactoacidosis, uraemia, alcoholism, salicylate poisoning, methanol or ethylene glycol poisoning
Inorganic phosphorus	< 0.32 mmol/L	Muscle atrophy, muscle pain, central nervous system symptoms such as disorientation, confusion, convulsion, coma, respiratory insufficiency with metabolic acidosis
	> 2.9 mmol/L	Tumour lysis syndrome, final stage of kidney failure
Bilirubin	> 257 µmol/L	Viral infections of the hepatobiliary tract
Glucose	< 2.5 mmol/L	Neuroglycopenic symptoms ranging from cognitive impairment to loss of consciousness
	> 27.8 mmol/L	Diabetic coma; osmotic diuresis; diabetic ketoacidosis (beta-hydroxy-butyrate > 5mmol/L, standard bicarbonate < 10 mmol / L)
Total calcium	< 1.65 mmol/L	Hypocalcemic tetanus
	> 3.50 mmol/L	Risk of hypercalcaemia, metabolic encephalopathy and gastrointestinal problems
Ionised calcium	< 0.78 mmol/L	Hypocalcemic tetanus
	> 1.60 mmol/L	Risk of hypercalcaemia, metabolic encephalopathy and gastrointestinal problems
**Parameter**	**Value**	**Comment**
Potassium	< 2.8 mmol/L	Neuromuscular symptoms; general weakness of skeletal musculature; complete paralysis; cardiac arrest. Changes in ECG
	> 6.0* mmol/L	Weakness of skeletal muscles can lead to paralysis of respiratory muscles
Chlorides	< 75 mmol/L	Metabolic alkalosis
	> 125 mmol/L	Primary metabolic acidosis or pseudohyperchloremia (bromide intoxication)
Creatinine	> 654 µmol/L	Acute kidney failure, e.g. in multiple organ failure or sepsis
Creatine kinase	> 1000 U/L	Depends on the patient population
Lactate	> 5.0 mmol/L	Type A hyperlactatemia caused by inadequate delivery of oxygen to the tissues.
Lactate dehydrogenase	> 500 U/L	Depends on patient population
Lipase	> 700 U/L	Acute pancreatitis
Magnesium	< 0.41 mmol/L	Paresthesia, cramp, irritability and athletic tetanus; cardiac arrhythmias together with hypokalaemia; arrhythmias are amplified by the action of digitalisation
	> 2.00 mmol/L	Reduced transmission of neuromuscular pulse; sedation, hypoventilation with respiratory acidosis, muscular weakness and decreased tendon reflex
Uric acid	> 773 µmol/L	Acute urethral nephropathy with tubular blockage and kidney failure
Sodium	< 120 mmol/L	Tonicity disorders caused by disturbances in ADH-thirst mechanism, water absorption or the kidney’s ability to concentrate or dilute urine
	>160 mmol/L	Central nervous system disorders; disorientation and increased neuromuscular susceptibility
High-sensitive troponin	15 ng/L	Myocardial infarction or unstable angina pectoris (values are matched to the test method used)
Free T4 Total T3	> 45 pmol/L > 46 nmol/L	Thyrotoxicosis. Possible causes include: Graves’ disease, trophoblastic tumour, hyper functional adenoma, toxic nodular soreness, and in rare cases excessive TSH formation
Urea	> 35.6 mmol/L	Acute kidney failure; unlike pre-renal and post-renal failure, there is no disproportionate increase in urea compared with serum creatinine
Osmolality	< 240 mOsm/kg H_2_O	Cellular oedema; increased cell volume; development of neurological psychiatric symptoms
	> 330 mOsm/kgH_2_O	Cellular water loss and intracellular increase of osmotic active substances not passing through the cell membrane; central symptoms and coma
Osmolality gap	> 10 mOsm/kg H_2_O	Intoxication with substances that increase plasma osmolality such as ethanol, methanol, ethylene glycol, isopropanol and dichloromethane
**Blood gases and acid–base balance**		
pCO_2_	< 2.5 kPa	Hyperventilation
	> 6.7 kPa	Hypoventilation
pH	< 7.2	Characteristic of strong decompressed acidosis or alkalosis. Values <7.2 and > 7.6 are life-threatening
	> 7.6	
pO_2_	< 5.7 kPa	Oxygen saturation of haemoglobin < 80%, which is life-threatening
Toxicology		
Digoxin Digitoxin	> 2.6 mmol/L > 52 nmol/L	Non-heart symptoms such as tiredness, muscular weakness, nausea, vomiting, lethargy, headache and heart symptoms such as sinus arrhythmias, bradycardia, and various AV block levels
Ethanol	> 3.5 g/L	Alcohol poisoning, coma

***** over 7 years

## APPENDIX 5.1 List of neonatal critical limits that should be communicated to the attending physician (*61*)

**Table:**

**Parameter**	**Value**	**Comment**
Bilirubin	> 239 µmol/L	First day of life, e.g. in haemolitic newborn disease; risk of kernicterus
C-reactive protein	> 5.0 mg/L	Neonatal sepsis
Glucose	< 1.8 mmol/L	Inherited metabolic disorders; hyperinsulinism due to mother’s diabetes mellitus. Glucose concentration < 1.3 mmol/L should be treated by parenteral glucose
	> 18.2 mmol/L	Urgently identify cause before additional testing
Haematocrit	< 0.330 (L/L)	Anaemia with inadequate delivery of oxygen to tissue
	> 0.710 (L/L)	Hyper viscosity of blood, high resistance in blood circulation
Haemoglobin	< 85 g/L	Risk of multi-organ failure, particularly with the combination of ischaemia and hypoxia.
	> 230 g/L	Abnormal flow kinetics (hyperviscosity) with increased heart rate
IgM	> 0.2 g/L	Concentration of IgM in umbilical cord blood may be associated with intrauterine infection.
Potassium	< 2.6 mmol/L	Neuromuscular symptoms with hyporeflexia and paralysis of respiratory muscles.
	> 7.7 mmol/L	Heart rhythm impairment, skeletal muscles weakness and respiratory paralysis
White blood cell count	< 5.0 x 10^9^/L > 25.0 x 10^9^/L	High risk of neonatal sepsis if the number of granulocytes is < 5.0 x 10^9^/L and > 25.0 x 10^9^/L
pO_2_	< 4.9 kPa	Oxygen saturation of haemoglobin < 85%
Platelet count	< 100 x 10^9^/L	Limit for newborns with birth weight < 2500 g is 50 x 10^9^/L

Cerebrospinal fluid

Increased number of cells

Leukocytosis, tumour cells

Glucose concentration is significantly lower in cerebrospinal fluid than in serum (< 2.2 mmol/L)

Lactate > 2.2 mmol/L

Existence of pathogenic bacteria in a Gram-stained smear or agglutination test

Urine

Very positive for glucose and acetone on a test strip

Erythrocyte cylinders or > 50% dysmorphic erythrocytes

Gross haemoglobinuria (without erythrocytes in microscopy analysis)

Drugs and other substances of abuse

Differential blood count

Leukaemic reaction

Suspected leukaemia

Suspected aplastic crisis

Sickle cells

Malaria parasites

## APPENDIX 6 Minimum sample storage conditions for traceability purposes (*69*-*72*)

**Table:**

**Sample type**	**Time and temperature**
Serum, plasma, whole blood samples, sedimentation, body fluids, aspirate	48 h at 4 °C
Whole blood (acid-base balance syringes)	24 h at 4 °C
Urine samples for quantitative and qualitative analysis	24 h at 4 °C
Stool for occult bleeding or sample solution The derived faecal suspensions in buffer required for retesting of equivocal results.	24 h at 4 °C two weeks at – 20 °C
Samples for analytical toxicology	48 h at 4 °C
Samples for drug analysis	48 h at 4 °C
Samples for pregnancy tests	48 h at 4 °C
Samples for coagulation tests	24 h at 4 °C
Samples for specialised coagulation test	24 h at - 20 °C
Samples for molecular diagnostics (DNA isolation)	10 years at - 20 °C
Smears of peripheral blood and body fluids	1 month
Aliquots of occasional search test*	24 h at 4 °C
Serum taken after accidental prick with a needle or contact with potentially infectious material	1 year at - 20 °C
Storage of sample aliquots sent to a referral laboratory or collaborative institution (until receipt of the report)	At - 20 °C
Test cards (*i.e.* faecal occult blood test cards, some point-of-care strips)	24 h at 4 °C
Samples for criminal investigations	As long as required for investigation at an appropriate temp.

*After the analysis.

## APPENDIX 7 Minimum storage conditions for archiving laboratory documentation

**Table:**

**Documentation**	**Storage time**
Primary copy of records in patient’s paper or electronic medical records	Depending on the institution’s policy, minimum 10 years
Laboratory records of the general laboratory programme	1 year
Laboratory records of the results of the specialised laboratory programme, including tests of addictive substances, toxic substances, tumour markers, electrophoresis and immunofixation images, graphical display of results	5 years
Laboratory records of the results of the subspecialist laboratory programme, including tests of metabolic diseases, hereditary diseases, genetic analysis	Permanent
Laboratory records of the results of subspecialist laboratory programme in biochemistry, haematology, immunochemistry	3 years
Laboratory records of all results of the point-of-care programme	1 year
Laboratory records of evaluation of quality and technical records including outdated tests, records of materials submitted to collaborative laboratories	1 year
Requests for laboratory tests from primary care facilities and hospitals, transport lists, worksheets, work logs	3 months
Other forms of laboratory administration (different protocols, forms, instructions); point-of-care management system documents conducted by the laboratory; results of internal quality control assessment	3 years
Outcomes of external quality control assessment; quality management system documents	5 years
Requests for laboratory tests from primary care facilities and hospitals, transport lists, worksheets, work logs	3 months
Laboratory documentation according to HRN EN ISO 15189:2012	5 years
Upon expiration of the recommended storage time, documents, especially those in paper form that contain any personal information about the patient, should be destroyed.	
